# Elevated ApoE, ApoJ and lipoprotein-bound α-synuclein levels in cerebrospinal fluid from Parkinson’s disease patients – Validation in the BioFIND cohort

**DOI:** 10.1016/j.parkreldis.2023.105765

**Published:** 2023-07-12

**Authors:** Wojciech Paslawski, Per Svenningsson

**Affiliations:** a Laboratory of Translational Neuropharmacology, Department of Clinical Neuroscience, Karolinska Institutet, Stockholm, Sweden; b Basic and Clinical Neuroscience, Institute of Psychiatry, Psychology and Neuroscience, King’s College London, London, United Kingdom

**Keywords:** Apolipoproteins, α-synuclein, Lipoproteins, Parkinson’s disease, Cerebrospinal fluid

## Abstract

**Background:**

The progressive accumulation, aggregation, and spread of α-synuclein (aSN) are common hallmarks of Parkinson’s disease (PD) pathology. The genotype of apolipoprotein E (ApoE) influences PD progression. Recently we found that aSN co-localize with apolipoproteins on lipoprotein vesicles. We reported an increased level of ApoE, ApoJ and lipoprotein-bound aSN in CSF from early PD patients compared to matched controls. We also found reduced plasma ApoAI in PD patients.

**Objective:**

In this study, we used the same approach on the BioFIND cohort to validate our previous results and extended the studies to examine correlations with ApoE genotype, demographic variables, clinical symptoms and other biochemical findings reported in the BioFIND cohort.

**Methods:**

For the assessment, we used Western-Blot (WB) technique for apolipoproteins measurements in CSF and plasma from PD patients and healthy controls. Further, for measurement of aSN bound to lipoproteins, we combined immunodepletion with the enzyme-linked immunosorbent assay (ELISA).

**Results:**

Levels of ApoE, ApoJ and lipoprotein bound aSN were significantly increased in CSF from PD patients compared to controls. We also observed decreased levels of ApoAI and ApoJ in plasma from PD patients compared to controls.

**Conclusions:**

Concluding, the present data validated our previous findings. Altered lipoproteins appear to be important in early PD pathology and may be involved in *mechanisms* underlying aSN cell-to-cell transfer in the nervous system and be developed in algorithms for early diagnosis of PD.

## 1. Introduction

Parkinson’s disease (PD) is, after Alzheimer’s disease, the second most common neurodegenerative disorder. Its diagnosis is based on a clinical examination and the presence bradykinesia together with rigidity and/or resting tremor [1]. PD is also associated with non-motor symptoms like depression, hyposmia, sleep disorders, cognitive impartment and constipation. PD is confirmed *post-mortem* by a loss of neuromelanin-containing dopaminergic neurons in the S*ubstantia nigra pars compacta (SNc)* and the presence of abnormal α-synuclein (aSN)-enriched inclusions called Lewy bodies (LBs). aSN is engaged in synaptic vesicle trafficking, exocytosis, regulation of neurite outgrowth and nerve cell adhesion, and its aggregates can damage cell membranes and spread from cell to cell [2]. Alterations in the levels of aSN species in cerebrospinal fluid (CSF) from PD patients have been reported, but the magnitude and/or reproducibility of reported changes vary considerably [3]. Lately, the main focus for aSN in PD research is placed on aSN seed amplification assay (SAA) [4], where PD CSF added to aSN monomers induce *in vitro* aggregation with high diagnostic specificity. In our recent work [5], we described aSN interaction with lipoproteins, and it is possible role in aSN pathology spreading. We also reported changes in apolipoproteins levels between PD patients and controls.

Apolipoproteins are components of lipoprotein particles, which are responsible for lipid homeostasis [6]. aSN and apolipoproteins both change membrane structure and modulate membrane curvature,. The major apolipoprotein in CSF are in order ApoE, ApoAI, ApoAII, ApoCs, ApoJ, and ApoD [7]. The ApoE level is high in the paravascular (glymphatic) space [8], where it also can co-localize with amyloidβ and help its clearance through the blood-brain barrier [9]. Neuronal ApoE levels are increased after cellular stress or damage and postulated to play a role in their repair [10]. ApoE allelic variants are associated with an increased risk for Alzheimer’s disease [11] and cognitive decline in PD [12]. While the ApoE genotypes are widely studied in neurodegeneration [13], less work has been performed at the protein level. Regarding other apolipoproteins ApoAI together with ApoE is responsible for lipid transportation and delivery in the brain. It has been also shown that the plasma level of ApoAI might serve as a good biomarker for PD [14]. For ApoJ it has been shown that LBs that contain a high amount of ApoJ have less aSN suggesting a protective role for ApoJ in PD [15]. Still, there is a lack of PD biomarkers that reflect ongoing brain pathology, and new discoveries are often not reproducible. Consequently, the present study aimed to validate our previous findings regarding changes in apolipoproteins and lipoprotein-bound aSN levels between PD patients and controls. For this purpose, we used the BioFIND study cohort [16], on which blinded analysis was performed. As a result, we were able to confirm our previous findings that ApoE, ApoJ and lipoprotein-bound aSN levels are significantly increased in CSF, and ApoAI decreased in plasma, from PD patients compared to controls.

## 2. Materials and METHODS

### Samples:

Samples used in the study originated from BioFIND (http://biofind.loni.usc.edu), an observational, multi-center, cross-sectional study of moderate-to-advanced PD participants [16]. Enrolled PD participants met the modified United Kingdom PD Society Brain Bank (UKPDBB) clinical diagnostic criteria that require all three classic motor signs of PD (tremor, bradykinesia, and rigidity) to be present for study enrolment. The characteristic of used cohort is present in Table 1. The institutional review board of BioFIND approved the study protocol. Written informed consent was obtained from each study participant.

### Data collection:

We included a range of data available from the BioFIND study. All data was directly extracted from the BioFIND database as of May 2022.

#### Demographics and patients characteristics:

Age, sex, education year, weight, height and temperature.

#### Clinical scores and medication:

Movement Disorder Society Unified PD rating scale (MDS-UPDRS), Schwab & England Activities of Daily Living Scale, Montreal Cognitive Assessment (MoCA), REM sleep behaviour disorder (RBD) Questionnaire, RBD phenotype and Levodopa Equivalent Dose (LEDD).

#### Biospecimen data:

Supine blood pressure (BP) – systolic, Supine BP – diastolic, Supine heart rate, Seating BP – systolic, Seating BP – diastolic, Seating heart rate, Standing BP – systolic, Standing BP – diastolic, Standing heart rate, Plasma WBC, Plasma RBC, Plasma haemoglobin (Hb), Haematocrit, Platelet Count, CSF WBC, CSF RBC, CSF Hg, Total glucose.

#### Genetic information:

The presence of the glucocerebrosidase (GBA) gene mutations. APOE allelic variants data, obtained from the AMP PD Knowledge Platform as of August 2022.

#### Other data:

CSF Aβ1–42, total Tau (t-tau), phosphorylated Tau (p tau), aSN and phosphorylated aSN. Range of lipidomics, metabolomics and proteomics [17–22] results available at BioFIND database (http://biofind.loni.usc.edu ).

### Chemicals and antibodies:

Unless stated otherwise, all chemicals were purchased from Sigma-Aldrich (Merck KGaA) and were of an analytical grade. All solutions were prepared using Milli-Q deionized water (Millipore). The list of antibodies, and methods in which they were used, can be found in Table S1.

### Sodium dodecyl sulphate polyacrylamide gel electrophoresis (SDS-PAGE):

The unbiased data analysis of all samples was achieved by receiving coded and randomized samples from the BioFIND cohort. Normalization samples, 3 CSF samples for CSF analysis and 3 plasma samples for plasma analysis, were run on each gel to normalize between each run. Plasma samples were diluted 10-fold in MQ water before proceeding to further steps. The sample was mixed in a 1:1 ratio with 20 mM TCEP, to reduce disulfide bridges, and then 1:1 with a denaturing loading buffer (50 mM Tris-HCl, 70 mM Tris, 1% lithium dodecyl sulphate (LDS), 5% glycerol, 0.25 mM EDTA, 0.11 mM SERVA Blue G-250, 0.0875 mM Phenol Red, pH-8.5) and boiled at 95 ^◦^C for 5min. Next, samples were separated using a 4–12% bis-tris acrylamide gel using MES running buffer (50 mM Tris, 50 mM 2-(N-morpholino)ethanesulfonic acid (MES), 0.1% SDS, 1 mM EDTA, pH-7.3). 20 μL of the sample was used.

### Western blotting (WB):

After SDS-PAGE, gels were incubated in a transfer buffer (25 mM Tris, 192 mM glycine, 30% methanol) and 0.45 μm pore size Immobilon-P^™^ PVDF membranes (Millipore) were pre-wetted in methanol. Gels were assembled with membranes and the transfer was performed using the Trans-Blot Turbo Transfer System (BioRad) according to manufacturer protocols. Next, membranes were dried and shortly fixed in 100% methanol. For detection of small proteins, in samples with a low total protein content, a mixture of 3% paraformaldehyde/0.01% glutaraldehyde in PBS was used as a fixative [23]. Between each incubation period, membranes were washed three times in Tris Buffered Saline (TBS, 20 mM Tris, 150 mM NaCl, pH-7.6) containing 0.1%(v/v) Tween20 (TBS-T). Membranes were blocked during 1 h incubation in 5% skim milk at RT. Next, membranes were incubated overnight at 4 ^◦^C with a primary antibody diluted 1:1000 (v/v) in 1% skim milk. Membranes were thereafter incubated for 2 h with appropriate HRP-conjugated secondary antibody (Dako) diluted 1:10000(v/v) in 1% skim milk at RT. The signal was developed by a Clarity^™^ Western ECL Substrate (Bio-Rad) and levels of visualized proteins were estimated by measuring band intensities with ImageJ [24] and normalized to the amount of total protein in the sample. A fluorescent scan was used to visualize the protein ladder.

### Co-Immunoprecipitation (co-IP)/Apo depletion:

Depletion of ApoE-positive vesicles from human CSF, and ApoE and ApoAI positive vesicles from human plasma was performed using the co-IP approach. 200 μL of CSF, or 200 μL of 10x diluted plasma, was used as a starting material. Protein A/G agarose beads (Abcam) were pre-treated with 1% bovine serum albumin, dissolved in PBS buffer (20 mM phosphate, 150 mM NaCl, pH-7.4), to block any unspecific interactions. The endogenous immunoglobulins were depleted from samples during 2 h incubation with protein A/G agarose beads, followed by elimination of beads via centrifugation. The antibody recognizing ApoE for CSF, and both ApoE and ApoAI antibodies for plasma, were added to the obtained solution and incubated overnight at 4 ^◦^C. Thereafter, samples were incubated for 6 h with protein A/G agarose beads at RT and spun. The beads were washed and the whole supernatant was collected for ELISA.

### aSN ELISA:

The aSN concentration before and after apolipoproteins depletion was measured using the LEGEND MAX^™^ Human α-Synuclein ELISA Kit (844101, BioLegend) according to the manufacturer’s instructions. Briefly, a microtiter plate pre-coated with the capture antibody was washed 4 times, samples were added after dilution in reagent buffer, and incubated overnight at 4 ^◦^C with shaking. The next day, the plate was washed 4 times, followed by the addition of the biotinylated detection antibody to each well and incubation for 2 h at room temperature (RT). The unbound detection antibody was removed by four washes. The streptavidin-HRP conjugate was added to each well, incubated for 1 h at RT and then the plate was washed four times to remove the remaining conjugate. Finally, the chemiluminescent substrate was added to each well and signals were read using a Tecan Spark 10 M plate reader (Tecan Nordic AB).

### Statistics:

Statistical analyses were performed using SPSS Statistics v26 (IBM) and GraphPad Prism software v9 (GraphPad Inc). Group comparisons for apolipoproteins and aSN bound to lipoproteins were performed using the Mann–Whitney *U* test after correcting for age and sex with a linear model. Other comparisons were performed using a Mann–Whitney *U* test and a Kruskal-Wallis H-test with a Dunn’s correction. A Spearman’s rank correlation coefficient was used to analyse dependence between two sets of data. The false discovery rate (FDR) Benjamini-Hochberg method was used to correct Spearman’s rank correlation p-values for multiple comparisons. All statistical tests were two-tailed and a p-value < 0.05 was considered statistically significant.

### Data availability:

The data is available in the BioFIND database (http://biofind.loni.usc.edu/).

## 3. Results

### 3.1. Apolipoproteins changes in CSF and plasma from PD patients

**Fig. 1.**
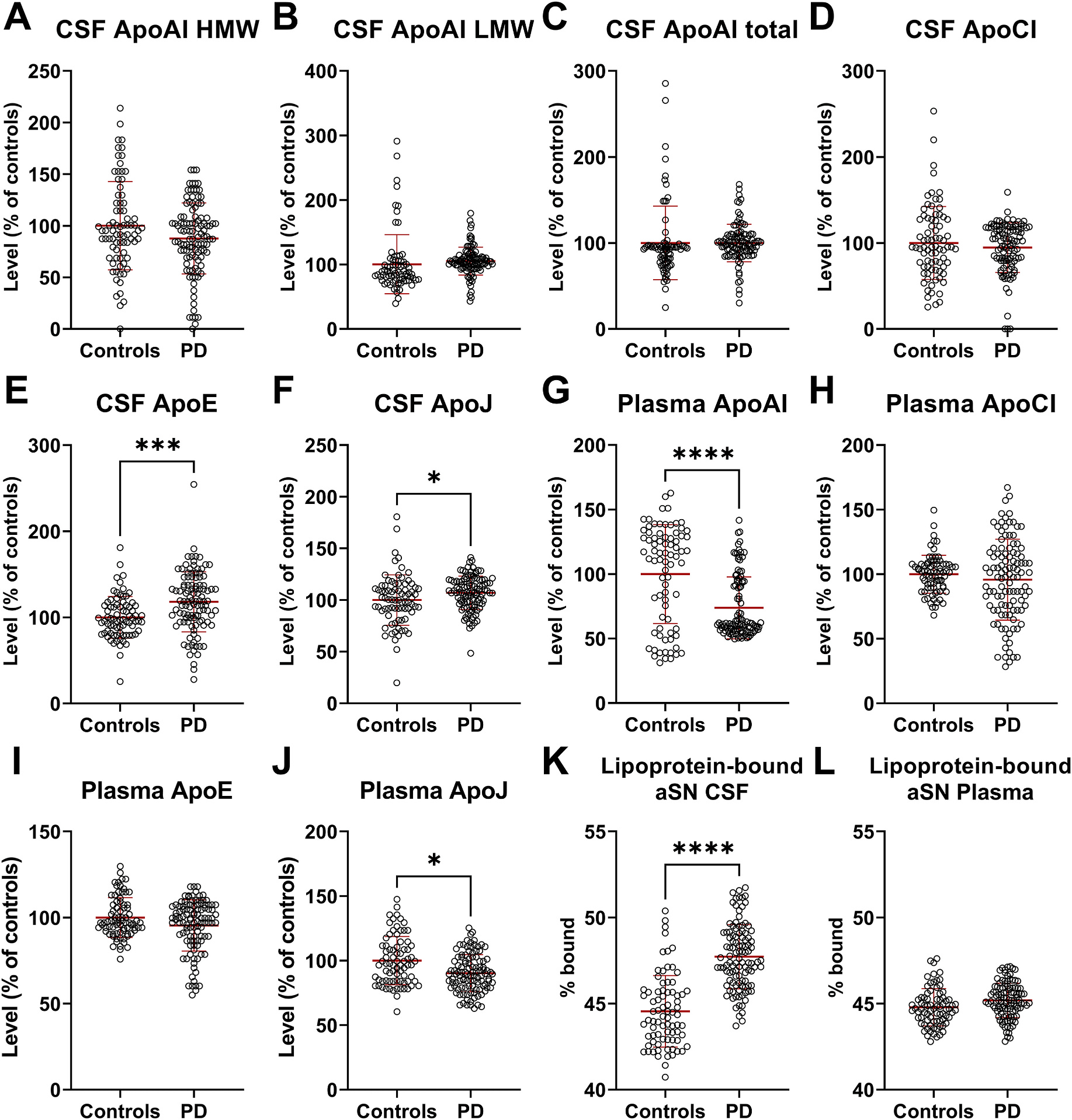
Alterations in apolipoproteins and lipoprotein bound aSN in CSF and plasma from the BioFIND cohort. The dot plots showing the alterations in CSF: HMW ApoAI **(A)**, LMW ApoAI **(B)**, total ApoAI **(C)**, ApoCI **(D)**, ApoE **(E)**, ApoJ **(F)**; Plasma: ApoAI **(E)**, ApoCI **(F)**, ApoE **(G)**, ApoJ **(H)**; and lipoprotein-bound aSN in CSF **(I)** and plasma **(J)**. The levels of apolipoproteins were normalized to the total protein levels in each sample. Group comparisons for apolipoproteins and aSN bound to lipoproteins were performed using the Mann–Whitney *U* test after correcting for age and sex with a linear model. *0.05 > p > 0.01, ***p < 0.001, ****p < 0.0001.

To analyse the changes between apolipoproteins in the BioFIND cohort, due to unmatched age (p = 0.0042) and sex (p = 0.0734) between controls and PD cases, we decided to use linear regression to correct for those variables in the analysis. We have not found significant changes in CSF ApoAI (Fig. 1A–C) and ApoCI (Fig. 1D). Considering ApoAI, we observed one interesting finding in CSF. While in our previous analysis we observed only one ApoAI reactive band for CSF samples, two bands were detected in many BioFIND samples (Fig. S1A). Still, we are not able to point to the origin of the second band, which for convenience; we named the High Molecular Weight (HMW) ApoAI band. Only one band was observed for other apolipoproteins (Figs. S1B–D). Considering other changes in CSF apolipoproteins, as in our previous work [5], we detected increased levels of ApoE (Fig. 1E, p = 0.0010) and ApoJ (Fig. 1F, p = 0.0423) in CSF from PD patients compared to controls.

For plasma apolipoproteins analysis, we were able to validate the decreased level of plasma ApoAI in the BioFIND cohort (Fig. 1G, p < 0.0001). No changes were observed for plasma ApoCI (Fig. 1H) and ApoE (Fig. 1I). Yet, compared to our previous work, we observed significantly decreased level of plasma ApoJ (Fig. 1J, p = 0.0163) in PD patients compared to controls.

### 3.2. Changes in lipoprotein bound aSN levels in plasma and CSF from PD patients and controls

Our previous observations suggest that around 40–50% of CSF aSN is associated with ApoE-positive lipoproteins [5]. To confirm this finding we performed a similar analysis on the BioFIND cohort and measured aSN levels before and after depletion of ApoE-positive vesicles in CSF and plasma (Fig. S2). In the BioFIND cohort the average of aSN bound to lipoproteins in controls is 44% and 48% in PD samples (Fig. 1K, p < 0.0001). These values are similar to our previous study, where we reported on average 40% of aSN bound to lipoproteins in controls and 47% in PD samples. We have not analysed plasma samples in our previous work. In the BioFIND cohort there was no change in aSN bound to lipoproteins between controls and PD plasma samples (Fig. 1L). Afterwards we performed receiver operating characteristic (ROC) analyses for significantly changed targets in both plasma and CSF and observed the highest diagnostic potential for aSN bound to lipoproteins in CSF (Fig. S3A, area under curve (AUC) 0.87), followed by CSF ApoE (Fig. S3B, AUC 0.69), plasma ApoAI (Fig. S3C, AUC 0.68), plasma ApoJ (Fig. S3D, AUC 0.64) and CSF ApoJ (Fig. S3E, AUC 0.61).

### 3.3. Correlations between apolipoproteins, lipoprotein bound aSN levels with demographic and clinical values

**Fig. 2.**
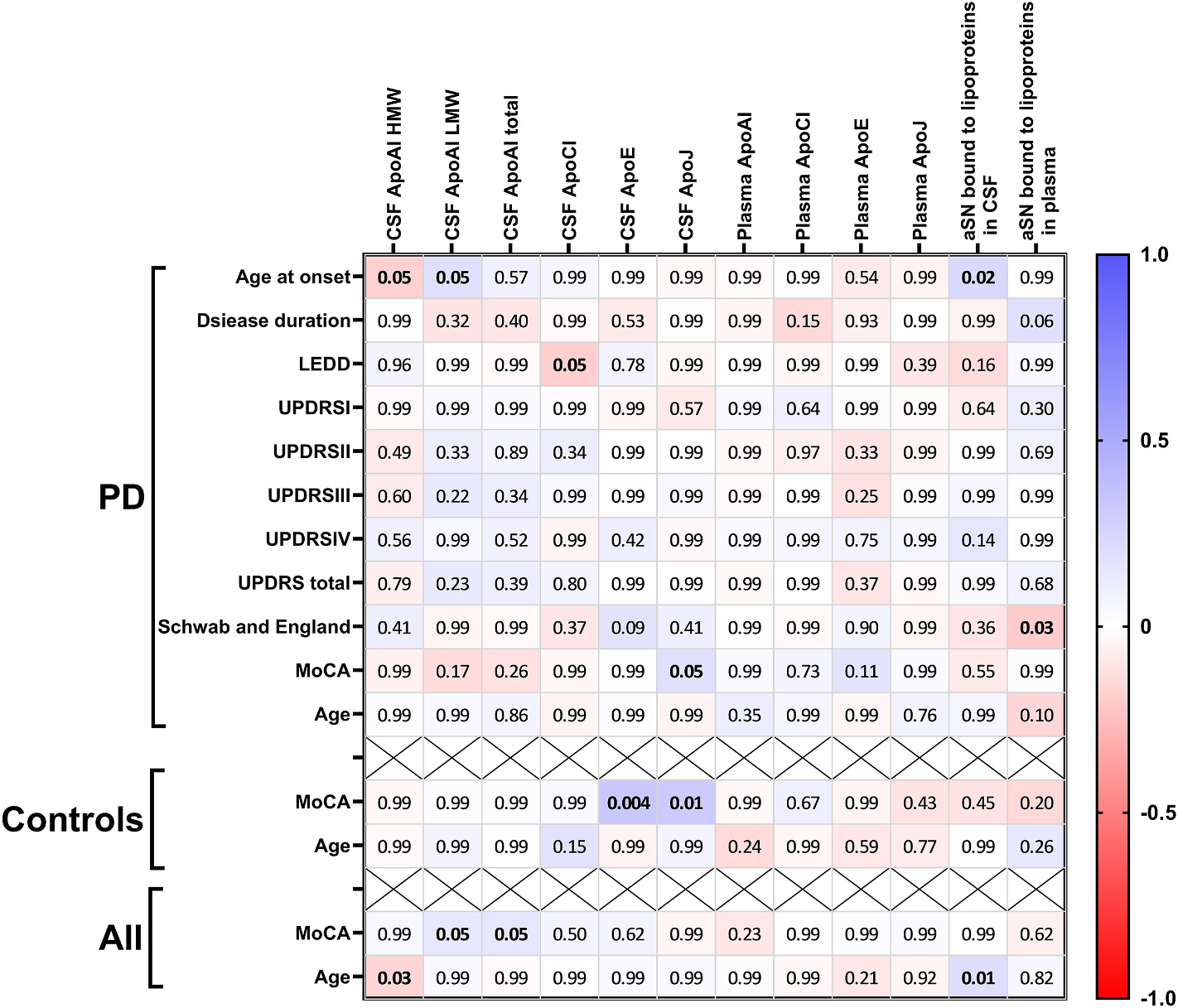
Association of analysed targets with clinical parameters in the BioFIND cohort. Heat map of Spearman’s rank correlation between clinical parameters and measured values. The p-values <0.05 are marked with a bold font.

Next, we examined if any of the analysed parameters correlate with available patient characteristics. When possible, the PD and control groups were divided to see if the correlation is specific for any of the analysed groups. In PD group CSF ApoAI LMW and lipoprotein-bound aSN were correlating with patients age at onset (Fig. 2, p = 0.05, r = 0.18 and p = 0.02, r = 0.22 respectively) and, on the other hand, CSF ApoAI HMW negatively correlated with age at onset (p = 0.05, r = − 0.18). CSF ApoCI correlated with LEDD score (p = 0.05, r = − 0.19) and CSF ApoJ with MoCA (p = 0.05, r = 0.18). Plasma lipoprotein-bound aSN was correlating with Schwab and England score (p = 0.03, r = − 0.21). In control group we observed association between CSF ApoE and ApoJ with MoCA score (p = 0.004, r = 0.33 and p = 0.01, r = 0.31 respectively). When both PD patients and controls were combined, we observed correlations between age and CSF HMW ApoAI (p = 0.03, r = − 0.16) and CSF lipoprotein-bound aSN (p = 0.01, r = 0.20). Also, both total and LMW CSF ApoAI correlated with MoCA score (p = 0.05 and r = 0.14 for both correlations).

For the other patients characteristics, we mostly observed correlations between plasma apolipoproteins and patients’ blood pressure, plasma red blood cell count, haemoglobin and haematocrit (Fig. S4).

### 3.4. Correlations between apolipoproteins and lipoprotein-bound aSN levels in plasma and CSF

Next, we investigated whether there is any correlation between levels of different types of apolipoproteins, or lipoprotein bound aSN, within and between plasma and CSF (Fig. S5). We observed moderate interactions between CSF ApoAI HMW and total CSF ApoAI (p < 0.0001, r = 0.47), CSF ApoE (p < 0.0001, r = 0.29), plasma ApoE (p = 0.005, r = 0.21) and lipoprotein-bound aSN in CSF (p = 0.0002, r = − 0.27). LMW ApoAI correlated with all other CSF apolipoproteins (p = 0.03, r = 0.17 for ApoAI HMW, p < 0.0001, r = 0.90 for ApoAI total, p = 0.004, r = 0.22 for ApoCI, p = 0.001, r = 0.24 for ApoE and p = 0.02, r = 0.18 for ApoJ) and CSF ApoE with CSF ApoJ (p <0.0001, r = 0.30). In plasma we observed correlation between all measured apolipoproteins (p < 0.0001 for all correlations, r 0.50–0.62).

### 3.5. Associations between apolipoproteins and lipoprotein bound aSN levels in plasma and CSF and other data deposited for the BioFIND cohort

In the last step, we examined if levels of analytes measured by us correlate or change with other data deposited for the BioFIND cohort.

#### 3.5.1. Genetic data

First, we examined if any of the apolipoprotein protein measures are affected by APOE allelic variant. APOE allelic variants were present for 153 samples with the following distribution E2/E2 (n = 2), E2/E3 (n = 16), E2/E4 (n = 2), E3/E3 (n = 101), E3/E4 (n = 30) and E4/E4 (n = 2). We have not observed significant differences, neither for CSF (Fig. S6) nor plasma (Fig. S7).

**Fig. 3.**
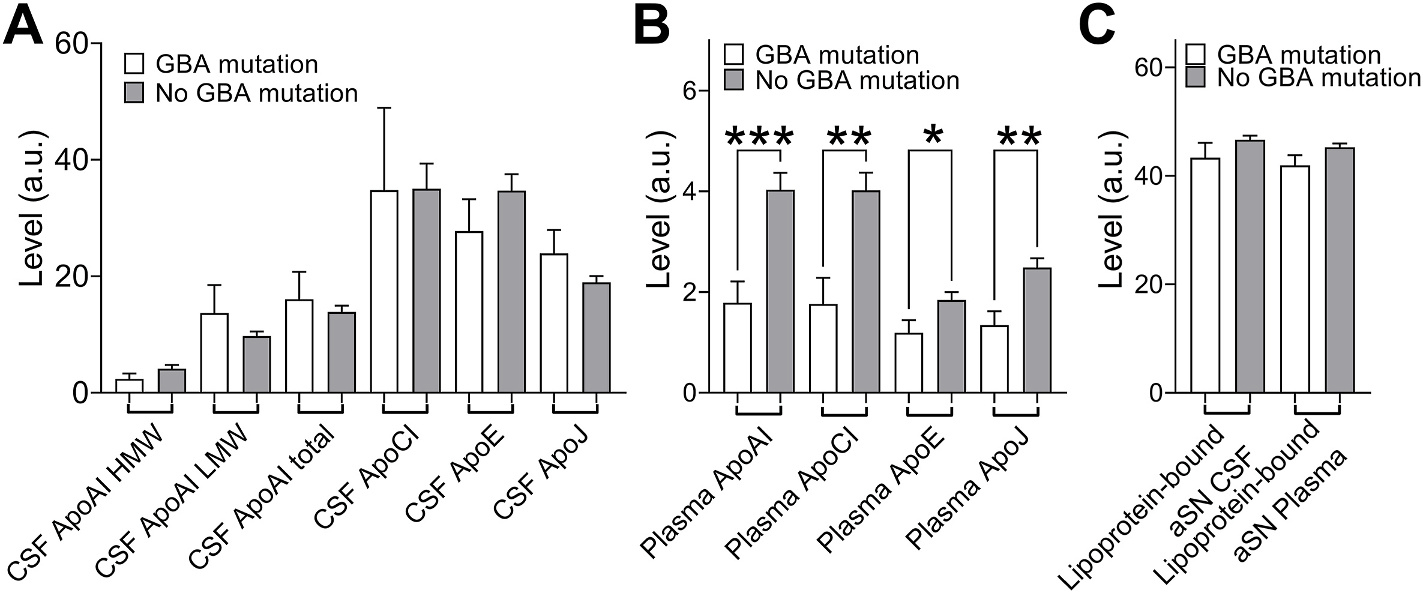
Effect of presence of GBA mutation on CSF and plasma apolipoproteins and lipoprotein-bound aSN. The plot showing the effect of GBA mutation presence on the CSF apolipoproteins **(A)** plasma apolipoprotiens **(B)** and lipoprotein-bound aSN **(C)**. Group comparisons were performed using the Mann–Whitney *U* test. *0.05 > p > 0.01, **p < 0.01, ***p < 0.001.

Then, we decided to examine if GBA mutations, collectively the single largest risk factor for the development of idiopathic PD [25], has an effect on any of the measured apolipoproteins, or the amount of aSN bound to lipoproteins. Following mutations of GBA were previously identified in the BioFIND cohort: N370S (n = 1), S196P; A456P (n = 1), M53V (n = 1), H255Q (n = 1), E388K (n = 1), Ex203A > G (n = 2), E326K (n = 4), dupG_84GG (n = 1), T369M (n = 2) and R257Q (n = 1). Due to a low number of individuals within each group, we decide to examine if any of the measured by us values is significantly changed in the combined GBA mutation group compared to individuals without mutation. We have not observed any changes for CSF apolipoproteins (Fig. 3A), but all plasma apolipoproteins were significantly lower in GBA mutation carriers group than rest of the cases (Fig. 3B, p = 0.0002 for ApoAI, p = 0.0012 for ApoCI, p = 0.0361 for ApoE and p = 0.0017 for ApoJ). We have not observed any changes in lipoprotein-bound aSN, neither in CSF nor in plasma (Fig. 3C).

#### 3.5.2. Metabolomics and proteomics data

Finally, we examined if apolipoproteins and lipoprotein-bound aSN levels correlate with omics data deposited for the BioFIND cohort. For clarity we focused only on correlations with p < 0.01 and for each analysed sample only the values measured in the same bio-fluid were considered (Table S2). Briefly, for CSF ApoAI, we observed correlations mostly with GPA lipids and sphingolipids. Therefore, ApoAI might be transporting those lipids between cells within the brain. CSF ApoCI was correlating with many classes of molecules and some proteins involved in electron transport and ATP biosynthesis. No clear pattern was observed for ApoE. ApoJ correlated with molecules important for glycolysis and glycolipid formation. Therefore, it suggests that CSF ApoJ might be important for these processes. Lipoprotein-bound aSN in CSF correlated with proteins that are located on exosomes and extracellular vesicles. No clear pattern was observed for plasma apolipoproteins. Interestingly, lipoprotein-bound aSN in plasma correlated with several plasma microRNAs.

## 4. Discussion

aSN possesses a membrane binding capacity and can interact with artificial lipid vesicles, cell membranes and extracellular vesicles. In our recent work, we also identified the interaction between aSN and apolipoproteins in the human CSF [5]. Likewise, it was reported that aSN can interact with lipoproteins also in the human plasma [26]. We also observed that approximately 45% of CSF aSN is bound to ApoE-positive lipoproteins, and this number is slightly higher in PD patients than in controls. Our analysis also demonstrated a significant increase in CSF ApoE and ApoJ, and a decrease in plasma ApoAI, in PD patients.

In the current work, we aimed to validate those findings in the BioFIND cohort. We were able to validate increased levels of ApoE, ApoJ and lipoprotein-bound aSN in CSF from PD samples compared to controls. We were also able to validate the decreased plasma ApoAI level and observed decreased levels of plasma ApoJ.

As we stated in our previous work [5], aSN-lipoprotein interactions, and their cellular uptake, raise a novel plausible explanation for the aSN uptake and spread in the brain. This hypothesis is further supported by recent work, which shows that neuronal ApoE regulates the cell-to-cell transmission of aSN [27]. Consequently, our observations suggest a functional involvement of ApoE, and lipoproteins themselves in PD pathology and/or defence against it. Apolipoproteins levels also correlated with proteins and compounds involved in important processes like lipid homeostasis, electron transport, ATP biosynthesis, glycolysis and glycolipid formation, which disturbance can also lead to cellular stress and neurodegeneration.

During our work we also discovered interesting observation regarding ApoAI. We revealed HMW species of ApoAI in the CSF, both in controls and PD patients. The HMW ApoAI band can be SDS-resistant ApoAI oligomers, caused by ApoAI amyloidosis or simply ApoAI accumulation. It can also indicate interaction (including amyloid type ones) with other proteins in the CSF, possibly even aSN oligomers and other prefibrillar species. However, our data do not allow us to speculate further on what type of HMW structure it is and further investigations are necessary to discover the phenomenon behind HMW ApoAI presence in CSF. Also, while CSF ApoAI is derived from blood [7], we did not observe correlation between plasma and CSF ApoAI. In this context, we need to consider that the central nervous system is sealed from the blood by the blood-brain and blood-CSF barriers. Both barriers present a number of active transport mechanisms expressed as a complex system comprising the highly specialized endothelial cells, pericytes, perivascular antigen-presenting cells, and astrocytes. Moreover, while we know that ApoAI is able to pass the barriers, partially due to clathrin-independent cholesterol-mediated endocytosis [28], we do not know all the mechanisms and the amount of ApoAI delivered to CSF. Moreover, we need to consider that portion of the ApoAI is accumulated in endothelial cell monolayers [28] and some is degraded within CNS as well. Therefore, while CSF ApoAI is derived from periphery, it does not imply that there is a correlation between plasma and CSF ApoAI levels.

Finally, it needs to be noted that the vast number of publications on ApoE are not focused on the ApoE protein, but on its gene. APOE genotype is associated with an increased risk for several diseases, including Alzheimer’s disease and synucleinopathies [29,30]. In our study, we observed no correlation between apolipoproteins level and its allelic variant, what is in line with previous observations showing no effect of APOE allelic variants on the PD development risk [30].

In summary, the increase of aSN on lipoprotein vesicles along with changes in apolipoproteins’ levels from PD patients compared to controls may be involved in cell-to-cell pathology progression in PD and should be considered in development of future diagnostics algorithms. Especially considering the fact that lipoproteins have recently been shown to decrease the amplification of αSN seeds in CSF SAA, and ApoAI and ApoE levels significantly correlated with SAA kinetic [31].

## Supplementary Material

1

## Figures and Tables

**Table 1:** Basic demographics and PD severity scores of studied subjects in the BioFIND cohort. Age (mean ± standard deviation), sex of PD patients and matched controls, and average severity scores (mean ± standard deviation) for PD patients.

Demographics			PD characteristics					
Diagnosis	PD	Control	Score	Value ((Mean ±SD)	Score	Value ((Mean ±SD)	Score	Value ((Mean ±SD)

**Age (Mean ±SD)**	67.80 ± 6.44	65.02 ± 7.15	**LEDD**	692.4 ± 382.4	**MDS-UPDRS I**	9.58 ± 5.69	**MDS-UPDRS total**	63.16 ± 21.48
**Number of cases**	108	78	**MoCA**	26.87 ± 2.47	**MDS-UPDRS II**	11.15 ± 6.47	**Schwab & England**	85.0 ± 9.52
**Males**	65	36	**Hoehn & Yahr**	N/A	**MDS-UPDRS III**	38.81 ± 13.35	**Disease Duration**	6.66 ± 3.26
**Females**	43	42	**Age at onset**	61.24 ± 6.39	**MDS-UPDRS IV**	3.62 ± 2.94		
