# Supplementary material for: Elevated ApoE, ApoJ and lipoprotein-bound α-synuclein levels in cerebrospinal fluid from Parkinson’s disease patients – Validation in the BioFIND cohort
Source: Parkinsonism Relat Disord. Author manuscript; Available in PMC 2024 May 31. (PMC11140586; doi:10.1016/j.parkreldis.2023.105765)
Supplement: 1 [file NIHMS1995748-supplement-1.pdf]

SUPPLEMENTRY MATERIAL

Elevated ApoE, ApoJ and lipoprotein-bound α-synuclein levels in cerebrospinal fluid from Parkinson's disease patients – validation in the BioFIND cohort

Wojciech Paslawski and Per Svenningsson

Table S1. Antibodies used in the study. Details of antibodies used in the present study.

	Description	Conjugate	Host	Clonality	Cat. No.	Distributor	Method
**Primary**	anti-ApoAI [EP1368Y]	NA	rabbit	monoclonal	ab52945	Abcam	**WB, immunodepletion**
	anti-ApoCI [EPR16813]	NA	mouse	monoclonal	ab198288	Abcam	**WB**
	anti-ApoE [E6D7]	NA	mouse	monoclonal	ab1907	Abcam	**WB**
	anti-ApoE	NA	goat	polyclonal	178479	EMD Millipore	**immunodepletion**
	anti-ApoJ [EPR2911]	NA	rabbit	monoclonal	ab92548	Abcam	**WB**
**Secondary**	anti-mouse IgGs	HRP	goat	polyclonal	P0447	Dako	**WB**
	anti-rabbit IgGs	HRP	goat	polyclonal	P0448	Dako	**WB**

Table S2. Correlation of apolipoproteins and lipoprotein bound aSN levels with omics data deposited for the BioFIND cohort. Significant correlations, with corresponding r and n values, between apolipoproteins and lipoprotein-bound aSN levels with omics data deposited for the BioFIND cohort. The values were corrected for multiple comparison using FDR method.

**CSF ApoAI**		**CSF ApoCI**		**CSF ApoE**		**CSF ApoJ**		**aSN bound to lipoproteins CSF**		**Plasma ApoAI**		**Plasma ApoCI**		**Plasma ApoE**		**Plasma ApoJ**		**aSN bound to lipoproteins Plasma**	
Homovanillic acid	p=0.0109<br>r=0.35<br>n=51	MIME	p=0.0097<br>r=-0.19<br>n=185	homoarginine	p=0.0081<br>r=-0.27<br>n=98	Bis(2-hydroxypropyl)amine	p=0.0057<br>r=0.26<br>n=114	SPRC	p=0.0089<br>r=-0.19<br>n=185	16-hydroxypalmitate	p=0.0046<br>r=0.28<br>n=98	4-acetamidobutanoate	p=0.0076<br>r=0.27<br>n=97	methionine sulfone	p=0.0070<br>r=-0.27<br>n=98	3-methyl-2-oxovalerate	p=0.0092<br>r=-0.26<br>n=98	miR-99b	p=0.0098<br>r=-0.27<br>n=89
sphingomyelin (d18:1/20:0 d16:1/22:0)	p=0.0103<br>r=0.26<br>n=96	COCH	p=0.0091<br>r=-0.19<br>n=185	5-Hydroxyindoleacetic acid	p=0.0073<br>r=0.25<br>n=113	S-methylcysteine	p=0.0033<br>r=0.32<br>n=81	CATD	p=0.0087<br>r=-0.19<br>n=185	gulonate	p=0.0039<br>r=0.30<br>n=92	4-acetamidophenylglucuronide	p=0.0067<br>r=-0.54<br>n=24	5-methylthioadenosine (MTA)	p=0.0062<br>r=0.27<br>n=98	N-acetylcitrulline	p=0.0070<br>r=0.30<br>n=80	1-palmitoyl-2-linoleoyl-GPI (16:0/18:2)	p=0.0081<br>r=0.27<br>n=98
Quinolinic acid	p=0.0080<br>r=0.37<br>n=51	VASN	p=0.0091<br>r=-0.19<br>n=185	Proline	p=0.0053<br>r=0.26<br>n=114	G3P	p=0.0028<br>r=-0.22<br>n=185	NCAN	p=0.0063<br>r=0.20<br>n=181	ergothioneine	p=0.0029<br>r=-0.30<br>n=98	ergothioneine	p=0.0029<br>r=-0.30<br>n=98			2'-deoxyuridine	p=0.0056<br>r=-0.45<br>n=36	dimethyl sulfone	p=0.0075<br>r=-0.27<br>n=98
sphingomyelin (d18:1/24:1 d18:2/24:0)	p=0.0061<br>r=0.28<br>n=98	CH3L1	p=0.0086<br>r=-0.19<br>n=185	3-hydroxybutyrate (BHBA)	p=0.0040<br>r=0.29<br>n=98	Aspartate	p=0.0018<br>r=0.29<br>n=113	CH3L1	p=0.0061<br>r=-0.20<br>n=185	2-keto-3-deoxy-gluconate	p=0.0029<br>r=0.30<br>n=98					adenosine 5'-diphosphate (ADP)	p=0.0038<br>r=0.41<br>n=47	Detyrosinated Tubulin	p=0.0071<br>r=0.34<br>n=61
1-palmitoyl-2-arachidonoyl-GPC (16:0/20:4n6)	p=0.0060<br>r=0.28<br>n=98	PEDF	p=0.0069<br>r=-0.20<br>n=185					A1AT	p=0.0053<br>r=-0.20<br>n=185	O-acetylhomoserine	p=0.0017<br>r=-0.36<br>n=72					4-methyl-2-oxopentanoate	p=0.0032<br>r=-0.29<br>n=98	miR-Let-7e	p=0.0071<br>r=-0.28<br>n=89
sphingomyelin (d18:2/24:1 d18:1/24:2)	p=0.0047<br>r=0.28<br>n=98	TRFE	p=0.0062<br>r=-0.20<br>n=185					N-methyl-pipecolate	p=0.0042<br>r=-0.43<br>n=43	ribonate	p=0.0014<br>r=0.32<br>n=98					4-vinylguaiacol sulfate	p=0.0006<br>r=-0.49<br>n=45	miR-9	p=0.0065<br>r=-0.29<br>n=89
N-acetylglycine	p=0.0010<br>r=0.33<br>n=98	ENOG	p=0.0044<br>r=-0.21<br>n=185															miR-155	p=0.0050<br>r=-0.29<br>n=89
1-stearoyl-2-linoleoyl-GPC (18:0/18:2)	p=0.0008<br>r=0.33<br>n=98	FBLN3	p=0.0042<br>r=-0.21<br>n=185															Tyrosinated Tubulin	p=0.0046<br>r=0.36<br>n=61
TNR21	p=0.0008<br>r=0.25<br>n=185	FAM3C	p=0.0041<br>r=-0.21<br>n=185															Taurourso-deoxycholate	p=0.0041<br>r=0.47<br>n=35
1-palmitoyl-2-linoleoyl-GPC (16:0/18:2)	p=0.0002<br>r=0.37<br>n=99	Aspartate	p=0.0039<br>r=0.40<br>n=51															miR-107	p=0.0040<br>r=-0.30<br>n=89
		DIAC	p=0.0032<br>r=-0.22<br>n=185															3-carboxy-4-methyl-5-propyl-2-furanpropanoate (CMPF)	p=0.0032<br>r=-0.29<br>n=98
		APOL1	p=0.0028<br>r=-0.22<br>n=185															B-Alanine	p=0.0018<br>r=0.23<br>n=185
		NRP2	p=0.0028<br>r=-0.22<br>n=185																

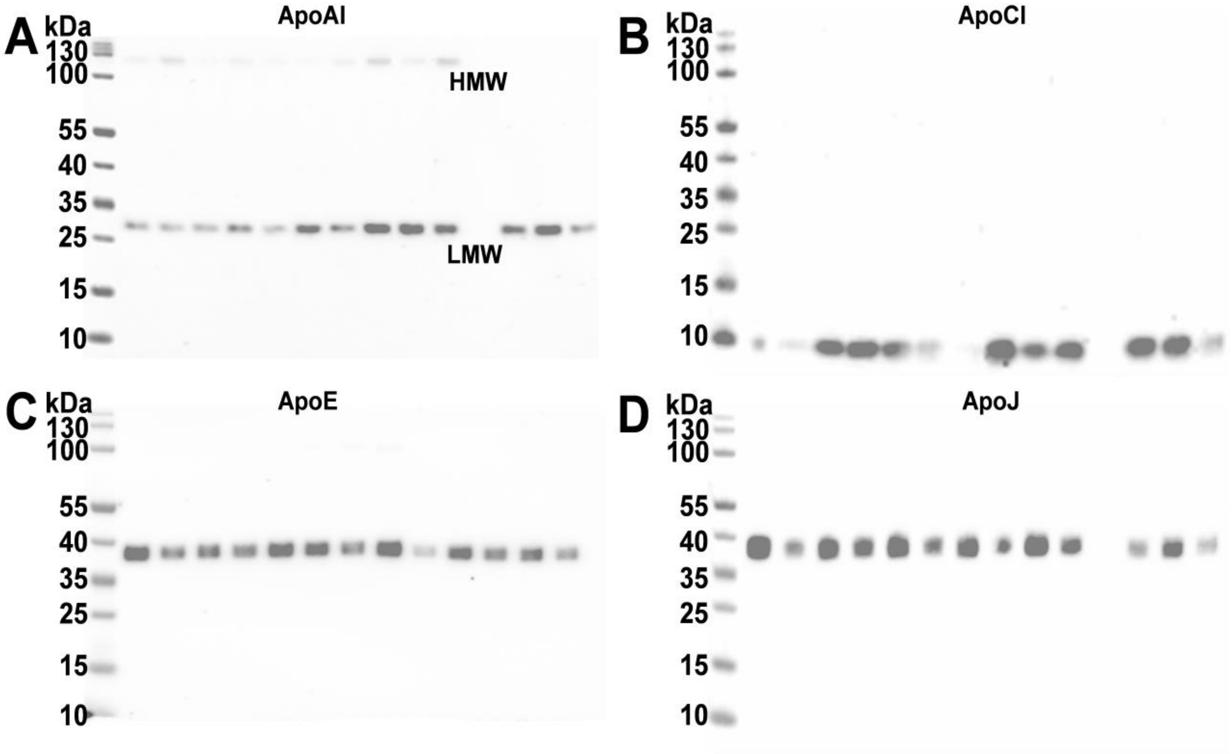

Figure S1. Apolipoprotein detection using the WB method. Example representative autoradiograms obtained after running CSF samples and performing WB procedure against ApoAI **(A)**, ApoCI **(B)**, ApoE **(C)** and ApoJ **(D)**. HMW – high molecular weight band of ApoAI. LMW – low molecular weight band of ApoAI.

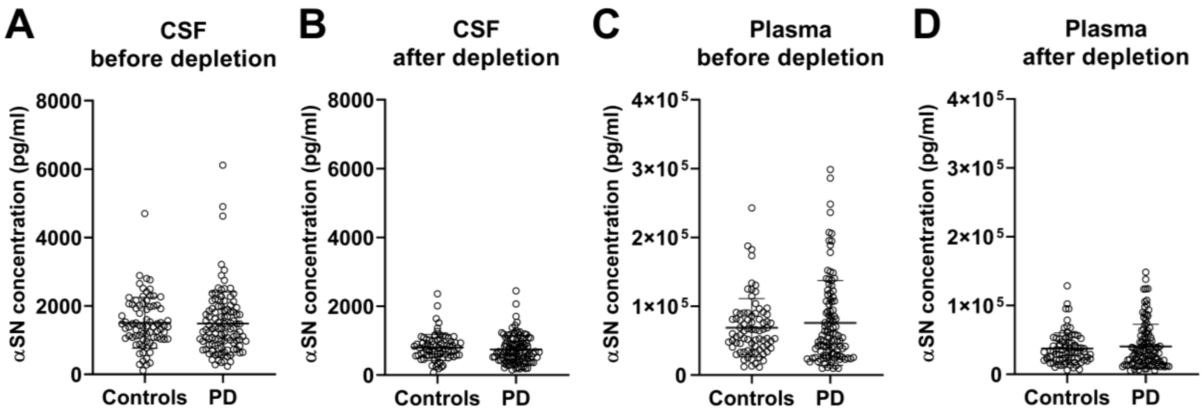

Figure S2. aSN levels before and after lipoprotein depletion. The dot plots showing the levels of aSN: in CSF before **(A)** and after **(B)** ApoE depletion; and in plasma before **(C)** and after **(D)** ApoE and ApoAI.

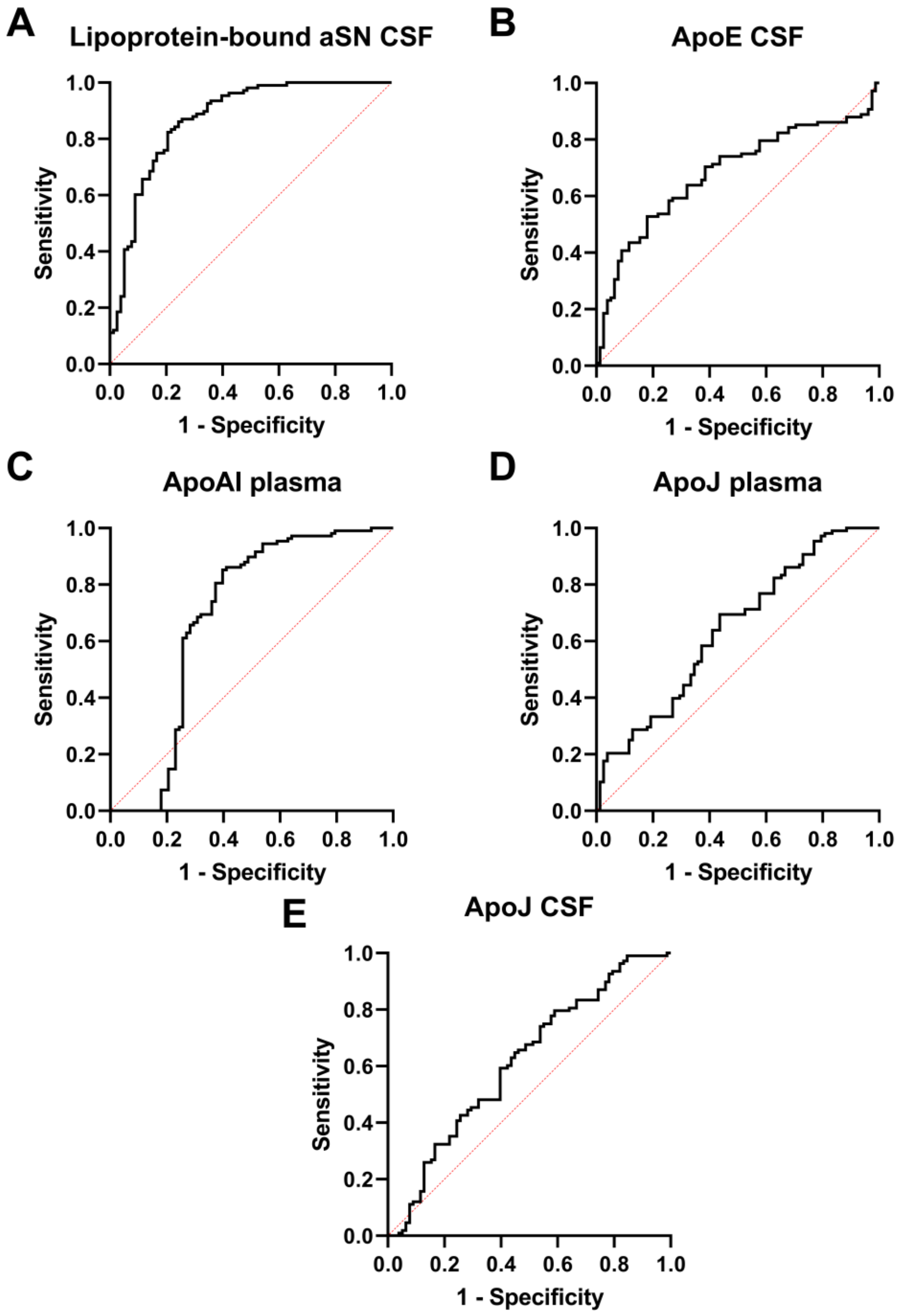

Figure S3. ROC curves. Age and gender adjusted ROC curves showing the relationship between sensitivity (true positive) and 1 − specificity (true negative) in determining the predictive value of: (A) lipoprotein-bound aSN, (B) CSF ApoE, (C) plasma ApoAI, (D) CSF ApoJ and (E) plasma ApoJ.

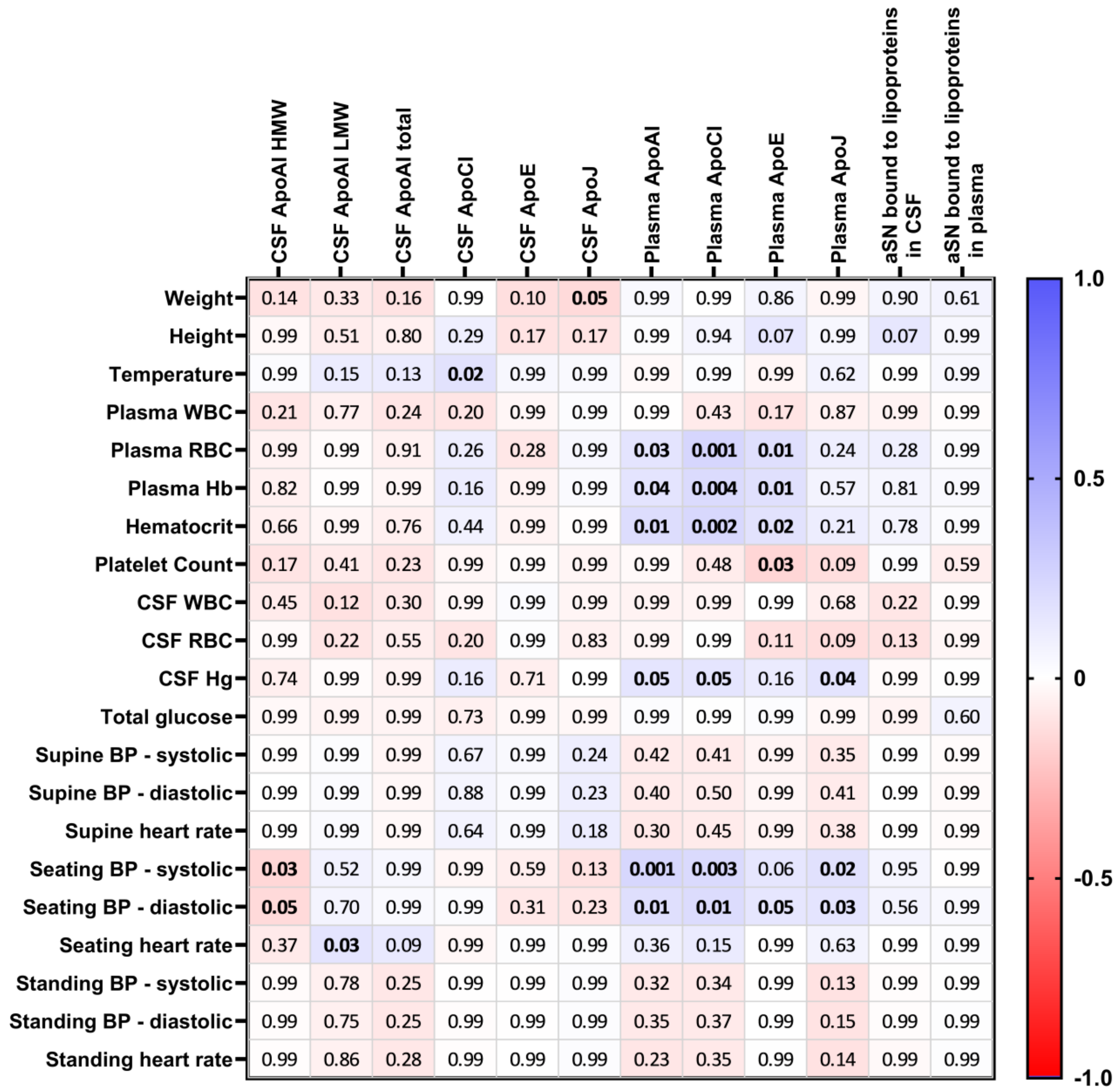

	CSF ApoAI HMW	CSF ApoAI LMW	CSF ApoAI total	CSF ApoCI	CSF ApoE	CSF ApoJ	Plasma ApoAI	Plasma ApoCI	Plasma ApoE	Plasma ApoJ	aSN bound to lipoproteins in CSF	aSN bound to lipoproteins in plasma
Weight	0.14	0.33	0.16	0.99	0.10	**0.05**	0.99	0.99	0.86	0.99	0.90	0.61
Height	0.99	0.51	0.80	0.29	0.17	0.17	0.99	0.94	0.07	0.99	0.07	0.99
Temperature	0.99	0.15	0.13	**0.02**	0.99	0.99	0.99	0.99	0.99	0.62	0.99	0.99
Plasma WBC	0.21	0.77	0.24	0.20	0.99	0.99	0.99	0.43	0.17	0.87	0.99	0.99
Plasma RBC	0.99	0.99	0.91	0.26	0.28	0.99	**0.03**	**0.001**	**0.01**	0.24	0.28	0.99
Plasma Hb	0.82	0.99	0.99	0.16	0.99	0.99	**0.04**	**0.004**	**0.01**	0.57	0.81	0.99
Hematocrit	0.66	0.99	0.76	0.44	0.99	0.99	**0.01**	**0.002**	**0.02**	0.21	0.78	0.99
Platelet Count	0.17	0.41	0.23	0.99	0.99	0.99	0.99	0.48	**0.03**	0.09	0.99	0.59
CSF WBC	0.45	0.12	0.30	0.99	0.99	0.99	0.99	0.99	0.99	0.68	0.22	0.99
CSF RBC	0.99	0.22	0.55	0.20	0.99	0.83	0.99	0.99	0.11	0.09	0.13	0.99
CSF Hg	0.74	0.99	0.99	0.16	0.71	0.99	**0.05**	**0.05**	0.16	**0.04**	0.99	0.99
Total glucose	0.99	0.99	0.99	0.73	0.99	0.99	0.99	0.99	0.99	0.99	0.99	0.60
Supine BP - systolic	0.99	0.99	0.99	0.67	0.99	0.24	0.42	0.41	0.99	0.35	0.99	0.99
Supine BP - diastolic	0.99	0.99	0.99	0.88	0.99	0.23	0.40	0.50	0.99	0.41	0.99	0.99
Supine heart rate	0.99	0.99	0.99	0.64	0.99	0.18	0.30	0.45	0.99	0.38	0.99	0.99
Seating BP - systolic	**0.03**	0.52	0.99	0.99	0.59	0.13	**0.001**	**0.003**	0.06	**0.02**	0.95	0.99
Seating BP - diastolic	**0.05**	0.70	0.99	0.99	0.31	0.23	**0.01**	**0.01**	**0.05**	**0.03**	0.56	0.99
Seating heart rate	0.37	**0.03**	0.09	0.99	0.99	0.99	0.36	0.15	0.99	0.63	0.99	0.99
Standing BP - systolic	0.99	0.78	0.25	0.99	0.99	0.99	0.32	0.34	0.99	0.13	0.99	0.99
Standing BP - diastolic	0.99	0.75	0.25	0.99	0.99	0.99	0.35	0.37	0.99	0.15	0.99	0.99
Standing heart rate	0.99	0.86	0.28	0.99	0.99	0.99	0.23	0.35	0.99	0.14	0.99	0.99

Figure S4. Association of analysed targets with other patients' parameters in the BioFIND cohort. Heat amp of Spearman's rank correlation between other patients' characteristics and measured values. Colour represent r-values (as presented on the right side scale), with corresponding p-values (insert text). The p-values <0.05 are marked with bold font. The values were corrected for multiple comparison using FDR method.

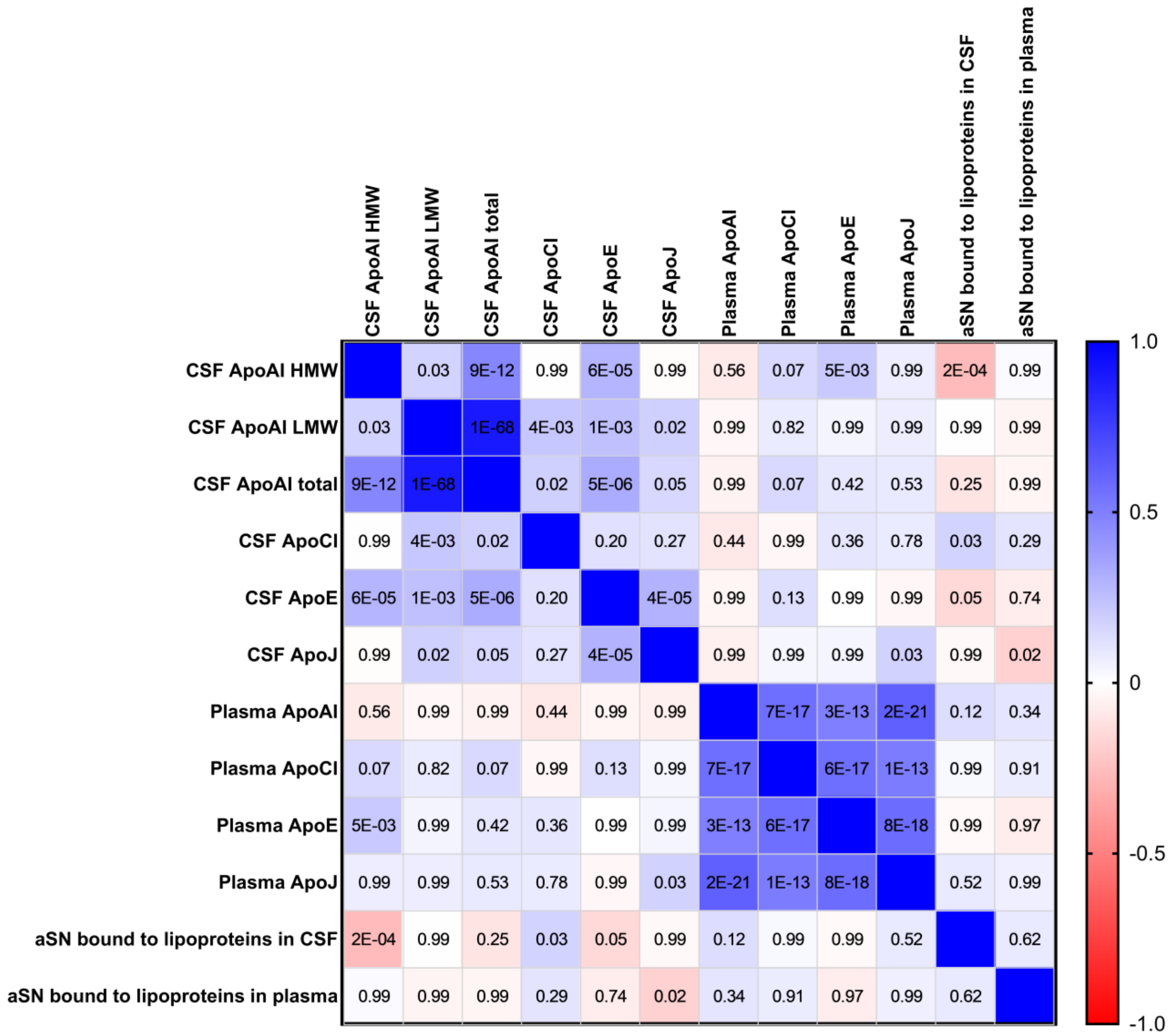

Figure S5. Correlation of apolipoproteins and lipoprotein bound aSN levels in both CSF and plasma samples from the BioFIND cohort. Heat map of correlations between apolipoproteins and lipoprotein bound aSN levels in both CSF and plasma samples. Colour represent r-values (as presented on the right side scale) with corresponding p-values (insert text). The p-values <0.05 are marked with bold font. The values were corrected for multiple comparison using FDR method.

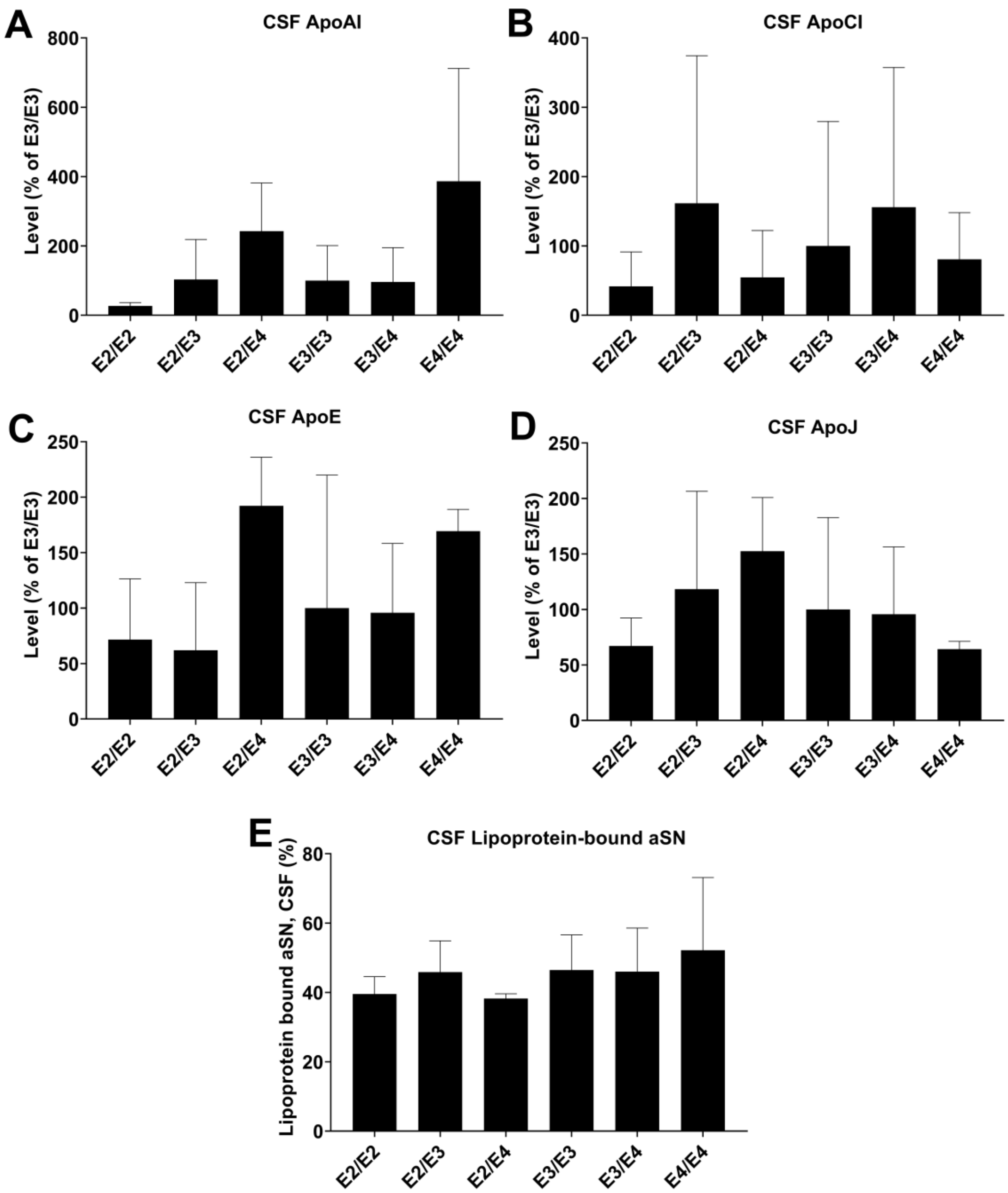

Figure S6. Effect of APOE allelic variant on CSF apolipoproteins and lipoprotein-bound aSN levels. The dot plot showing the effect of APOE allelic variant on the levels of: CSF ApoAI **(A)**, CSF ApoCI **(B)**, CSF ApoE **(C)**, CSF ApoJ **(D)**, aSN bound to lipoproteins in PD and controls CSF **(E)** and aSN bound to lipoproteins in other dementia cases **(F)**.

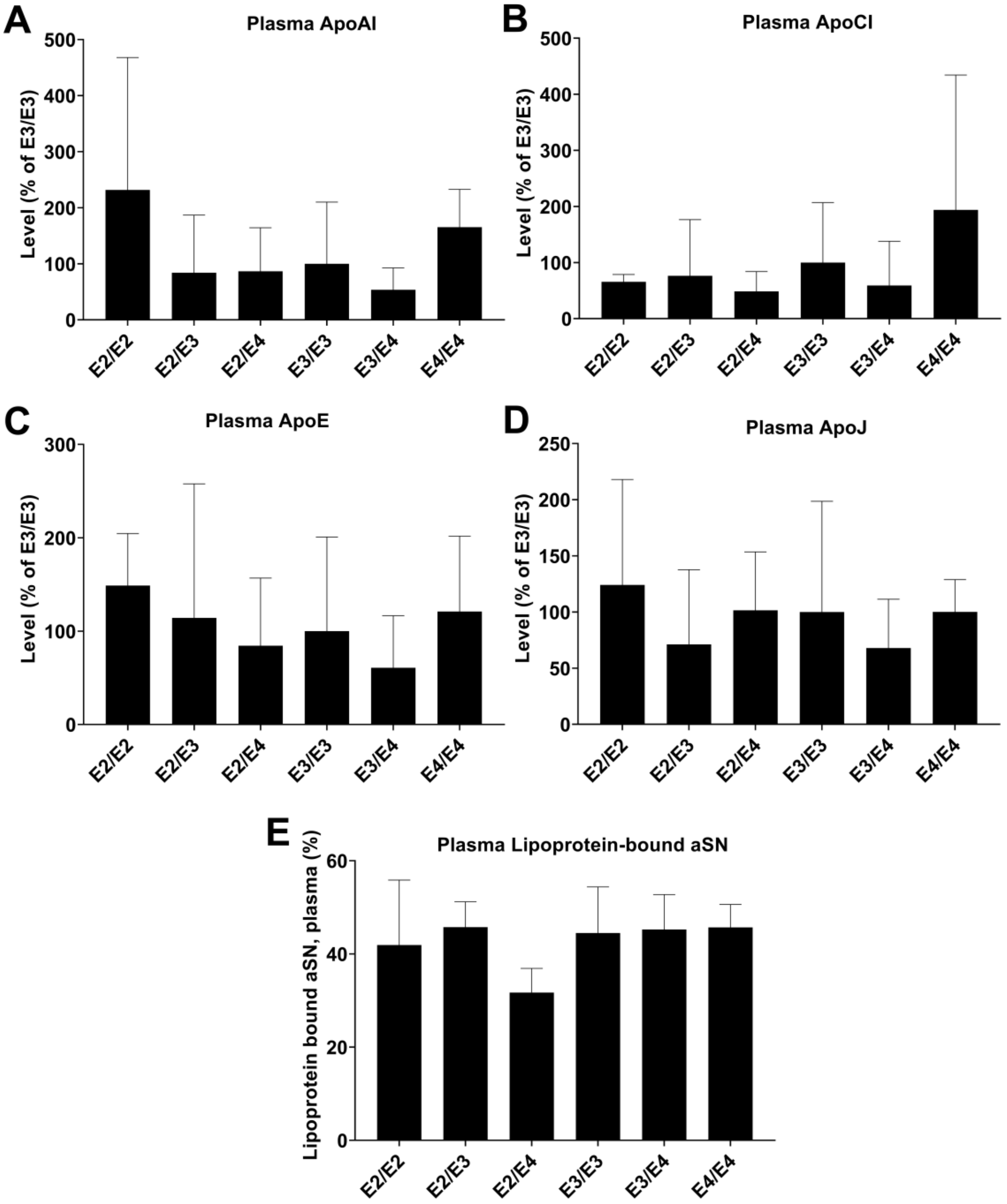

Figure S7. Effect of APOE allelic variant on plasma apolipoproteins and lipoprotein-bound aSN levels. The dot plot showing the effect of APOE allelic variant on the levels of: Plasma ApoAI **(A)**, Plasma ApoCI **(B)**, Plasma ApoE **(C)** Plasma ApoJ **(D)** and aSN bound to lipoproteins in PD and controls CSF **(E)**.
